# Systematic review: cultural adaptation and feasibility of screening for autism in non-English speaking countries

**DOI:** 10.1186/s13033-018-0200-8

**Published:** 2018-05-08

**Authors:** Turkiya S. Al Maskari, Craig A. Melville, Diane S. Willis

**Affiliations:** 1 Nursing & Healthcare School, College of Medical Veterinary and Life Sciences, University of Glasgow, 59 Oakfield Avenue, Glasgow, G12 8LL UK; 2 Institute of Health and Wellbeing, College of Medical Veterinary and Life Sciences, University of Glasgow, 1st Floor, Admin Building, Gartnavel Royal Hospital 1055 Great Western Road, Glasgow, G12 0XH UK; 3 Faculty of Health, Life and Social Sciences, Edinburgh Napier University, Sighthill Campus, Edinburgh, EH11 4BN UK

## Abstract

**Background:**

Screening children for autism has gained wider acceptance within clinical practice, and early intervention has improved outcomes. Increasingly, adapting an existing screening instrument is a common, fast method to create a usable screening tool, especially for countries with limited resources and/or expertise. However, concerns have been raised regarding adaptation adequacy and the feasibility of screening across cultural groups. This study systematically examined the levels of cultural adaptation and feasibility aspects considered when screening for autism in non-English speaking countries to build upon the sparse knowledge that exists on this topic in the literature.

**Methods:**

Nineteen studies, obtained from five electronic databases, were examined. PRISMA guidance was used for this review. The Ecological Validity Framework model, and Bowen Recommendations for Feasibility were adopted to extract relevant data, which was synthesised narratively.

**Results:**

Cultural adaptation within the included studies mostly involved language translation with little information offered to enable conclusions on how the processes were guided and maintained. Few cultural adjustments involved modifying screening methods; clarifying difficult concepts and changing instrument content were employed to address the core values, competence, beliefs, and norms of the adapted culture. However, less attention was given to adapt the screening goals within the context of cultural values, and customs or to consider interactional match between the clients and assessors. The review also highlighted an acceptable level of practicality to screen for autism but did not encourage integrating autism screening within routine practice or beyond the study context for different cultures.

**Conclusion:**

Concurring with previous literature, we agree that knowledge on cultural adaptation for autism screening instruments is limited and not sufficiently documented to establish adaptation levels (process and/or contents), and prove adequacy. However, this review provides an infrastructure to improve future adaptation processes. Integrating autism screening as routine medical practice is not encouraged and warrants further feasibility studies to minimize wasted resources and improve screening effectiveness in various health care systems.

## Background

The prevalence of autism is growing worldwide, precipitating the need for early intervention to improve outcomes [1]. Early intervention has increased interest in early screening [2, 3]. Global attention has turned to developing a screening instrument to facilitate early identification and diagnosis of children with autism younger than 30 months old [3–8]. This has resulted in a number of useful instruments that are classified into two levels. Level 1 instruments were designed to screen all children, regardless of their risk level of autism, and were used at the population level to support the identification process during the early stages of life and to boost outcomes resulting from early identification [9–13]. Level 2 instruments were intended to differentiate between ASD and other developmental disabilities [9–13]. They were aimed at those demonstrating high-risk features, such as children who had failed an autism-specific screening instrument; younger siblings of children who had been diagnosed with autism, and those who had a congenital (preterm status) or genetic (e.g., Fragile X, Down syndrome, or Angelman syndrome) conditions. Both types of instruments focused on specific questions and aided decision making within the referral and evaluation procedure [14].

Most research on screening instruments has been conducted in Western, English-speaking industrialised countries, where it is recognised that cultural issues impact this process [15–18]. Disparities in parental reporting, availability, or lack, of services for ethnic minorities, socioeconomic status and heterogeneity were noted as potential issues hindering the screening process [19, 20]. Literature supports using a valid and reliable screening test that considers the cultural context of a country [21–23]. Effective cultural adaptation can strengthen screening programmes, promote instrument validity at a conceptual level across different cultures, increase confidence in outcomes [24, 25] and guide future work. Increasingly, adapting an existing screening instrument to the population to be screened is becoming a common, quick and efficient method to provide a valid screening tool. This is especially true for those with limited resources or expertise in the field.

A growing body of research shows attempts to adapt various screening instruments for autism across cultural groups. However, cultural adaptation is recognised as a complicated and challenging process that goes well beyond language translation, and involves careful consideration of cultural values, customs and traditions, using appropriate knowledge and skill. There is no one right way to adapt the cultural validity of an instrument. The literature often suggests adaptation of culture through content and/or process, to increase congruence between the client’s ethno-cultural view and the adapted intervention [25, 26]. Content adaptation requires a deeper structural change, to address the core values, competence, beliefs and norms that match, both the investigator, and the targeted participants [25]. Process adaptation is usually considered a surface change and involves minor modification to interventions, such as literal language translation and changes in ethnicity in intervention materials [25, 26]. Consistently, the literature debates whether to undertake both levels of adaptation or to achieve adequate adaptation through the use of surface modification only [23, 24, 27, 28]. The most frequently advocated approach was to justify the use of a required level of adaptation through confirming the availability of data and resources (i.e. cost, expertise). This allowed for the proper testing of the cultural validity of the screening instruments [23].

In the field of autism screening, the adaptation process was insufficiently documented to justify its validity, and adaptations were limited to linguistic revision and surface modifications [29]. The level of adaptation that requires deep structural changes and incorporates cultural values within the adapted instrument were not investigated. Examining the content level of adapting an autism screening tool may extend existing knowledge of cultural adaptation on screening and guide the validation of future cultural adaptation work.

Little information is also available to justify integrating autism screening within routine practice. Although the feasibility of autism screening is not the principle aim of this study, the researchers thought shedding light on this important aspect was an appropriate secondary goal, to build upon the sparse knowledge that exists on this topic in the literature. In addition, information on the feasibility of a newly introduced procedure was thought to be relevant in improving, refining and adapting screening processes [30]. Bird, Le Boutillier [31] also indicated the importance of recognising “what is and is not feasible” within a practice, to minimise wasted resources, inform and prioritise decisions and improve effectiveness in health care systems.

To sum up, this review aims to determine the extent to which content level of adaptation is considered when screening for autism in non-English speaking countries. It also highlight the feasibility aspects involved in screening for autism in non-English speaking countries.

## Methods

The review followed the guidance in the PRISMA statement for reporting systematic reviews and meta-analysis [32].

### Eligibility criteria

All publications relating to the screening of children under 7 years of age for autism in non-English speaking countries were examined for this review. Only studies that used level I screening instruments and described relevant aspects of cultural adaptation, such as translations and/or cultural modifications, were included. No limitations on publication type or study design were imposed to ensure an adequate number of studies were identified.

### Information sources

Five databases, reflecting the topic, were selected: Psych INFO (EBSCOhost), MEDLINE (Ovid), CINAHL (EBSCOhost), EMBASE (Ovid), and ERIC (ProQuest); hand searches were also undertaken. Autism search terms were combined with ‘screening’ and ‘culture’. In March 2016, the first search was conducted. Reference lists of key studies and other reviews were scanned for potentially relevant articles. Google Scholar was also used as a source of ‘grey literature’. An updated search was done September 5, 2017. Examples and results from the literature search are provided in Table 1.

**Table 1:** Examples from the literature search

Search ID#	Search terms	Results
S18	S16 AND S17	(36)
S17	nurs* or allied health or health care provider	(138,950)
S16	S14 AND S15	(886)
S15	cultur*	(295,498)
S14	S7 AND S13	(25,630)
S13	S8 OR S9 OR S10 OR S11 OR S12	(1,257,837)
S12	assess*	(628,678)
S11	detect*	(110,318)
S10	test*	(740,387)
S9	surveillance	(0)
S8	screen*	(76,978)
S7	S1 OR S2 OR S3 OR S4 OR S5 OR S6	(70,399)
S6	rett*	(1752)
S5	kanner*	(810)
S4	pervasive*	(14,573)
S3	asperger*	(3496)
S2	ASD spectrum disorders	(32,945)
S1	autis*	(62,874)

Psych Info (36) 22/03/2016

### Study selection

Search results were imported into Endnote software X7.7, which was used to remove duplicates. Initially, only the title and abstract of each study were scrutinised for relevance independently by two reviewers (TAM, CM). Then the full text of eligible papers was retrieved if it met inclusion criteria or if the abstract did not provide adequate detail to warrant rejection. The full text of the paper was examined by the same two reviewers’ independently. The reviewers disagreed on 10 studies that failed to meet the age criteria (children exceeding 7 years of age). Through discussion agreement was reached to include six studies because more than 50% of their participants were under 12 years of age. Another eight papers were questionable due to lack of detail on the process of cultural adaptation; After discussion four papers that consider language translation were included.

### Extracted data

Three forms were developed to extract the relevant data: (1) a study characteristic form, (2) a contents level of adaptation form and (3) a feasibility form. The intention of the first form was to provide general information regarding the included studies, such as: author(s), country of publication, participant’s age group, assessor(s) and demonstrated screening instrument involved (see Table 2).

**Table 2:** Study characteristics

Author	Study design	Place	Participants	Informants	Screening tool
1. Albores-Gallo et al. [50]	Case control	Mexico Clinical/Psychiatric unit Community/Nurseries	N = 456 18–72 months Mean age = 4.46 years	Parents	The Mexican Modified Checklist for Autism in Toddlers (MM-CHAT) Detect nonverbal children with low function autism
2. Ben-Sasson and Carter [13]	Cohort	Israel Day care	N = 471 Age M = 12.7 months	Mainly mothers	The First Year Inventory (FYI)
3. Beuker et al. [51]	Cross sectional	Norway Hospital	N = 12,984 18 months Mean age: 18.53 months	Mothers	The Norwegian Modified Checklist for Autism in Toddlers (M-CHAT) Norwegian Early Screening of Autistic Traits (ESAT)
4. Canal-Bedia et al. [53]	Stage 1: Case control	Spain Extended Health Centre & Psychiatric	N-2480 18–36 months	Parents	The Spanish Modified Checklist for Autism in Toddlers (M-CHAT) + M-Chat Phone interview
	Stage 2: Cross sectional		N-2055 18–36 months		
5. Carakovac et al. [56]	Case control	Serbian Primary Healthcare & Psychiatric	N = 148 Mean age = 22.25–23.53 months	Parents	The Serbian Modified Checklist for Autism in Toddlers, Revised with Follow-Up (M-CHAT-R/F)
6. Fombonne et al. [49]	Case control	Mexico Autism Developmental Disorder Clinic Public primary school	N = 563 4–13 years Mean age: 8 years	Parents and teachers	Spanish version of the Social Responsiveness Scale (SRS)
7. Kamio et al. [46]	Cohort	Japan Routine check-up local Health Centre	N = 2516 18 months–3 years Mean age: 18.6–19.2	Parents	The Japanese version of the Modified Checklist for Autism in Toddlers (M-CHAT JV)
8. Kamio et al. [47]	Cohort	Japan Routine check-up local Health Centre	N = 1851 18 months–3 years Mean age: 18.7 months	Parents + trained interviewers	The Japanese verson of the Modified Checklist for Autism in Toddlers (M-CHAT JV) + Follow up Interview (FI)
9. Kara et al. [57]	Cross sectional	Turkey Well-child Paediatric	N = 191 18–36 months Mean age: 27.15	Parents	The Turkish version of the Modified Checklist for Autism in Toddlers (M-CHAT) + FI
10. Kara et al. [57]	Case control	Turkey Well child Paediatric Psychiatric-autism centre	N = 618 18–36 months Mean age: 27.15	Nurses and psychologists	The Turkish version of the Modified Checklist for Autism in Toddlers (M-CHAT)
11. Kondolot et al. [55]	Cross sectional	Turkey Family Health Centres	N = 4000 18–30 months Mean age: 23–24 months	Trained interviewers	The Turkish version of the Modified Checklist for Autism in Toddlers (M-CHAT)
12. Mohamed et al. [43]	Cross sectional	Egypt Primary Health Centres	N = 5546 1–2.9 years Mean age: 1.7	Parents	An Arabic validated version of Modified Checklist for Autism in Toddlers (M-CHAT)
13. Mohammadian et al. [45]	Case control	Iran Hospital nursery Psychiatric Hospital Autism Centre	N = 100 Mean age: 27.1 29.62 months	Mothers	The Iranian version of the Quantitative Checklist for or Autism in Toddlers (Q-CHAT)
14. Nygren et al. [54]	Cohort	Sweden Child Health Centre 2.5 year check-up	N = 3999 Mean age: 1.5 years	Mothers + trained nurses	The Sweden version of the Modified Checklist for Autism in Toddlers (M-CHAT) +FI + Joint Attention Observation (JA-OBS)
15. Perera et al. [53]	Cross sectional	Sri Lanka Primary Health Centre	N = 374 18–24 months	Mothers	Red Flag criteria + the Modified Checklist for Autism in Toddlers (M-CHAT)
16. Perera et al. [19]	Case control	Sri Lanka Paediatric Hospital	N = 105 18–48 months Mean age: 36–40 months	Mothers	The Pictorial Autism Assessment Schedule (PAAS)
17. Samadi and McConkey [7]	Cohort	Iran Population based Kindergarten and Pre-school Centres	N = 2941 2–5 years	Parents	Hiva + follow-up interview (FI) + the Modified Checklist for Autism in Toddlers (M-CHAT)
18. Seif Eldin et al. [42]	Case control	9 Arab countries Not reported	N = 228 18–36 months	Parents	The Modified Checklist for Autism in Toddlers (M-CHAT)
19. Seung et al. [48]	Cohort	Korea Day care, public HC, Hospitals, paediatric clinic	N = 2300 16–36 months	Parents + first author for FI	The Korean Modified Checklist for Autism in Toddlers (K-M-CHAT)-2 + Phone FI
20. Wong et al. [44]	Case control	Hong Kong Maternal and child Health clinics Psychiatric	N = 212 13–86 months	Parents + trained investigator	Checklist for Autism in Toddlers (CHAT-23)

+ Data reported, − data not reported

The second form was adapted from the Ecological Validity Framework (EVF) [33] model, to examine the extent of content level adaptation across the studies investigated within the review. The EVF was adopted because it is one of the first and most widely cited frameworks used to identify the critical elements in which cultural adaptation can play a role, and to address both surface and deep-level adaptations [34]. The model highlights effectiveness in a number of studies, when used to adapt culturally sensitive treatments [35–38]. This model has eight dimensions which focus on: language, metaphors, person, contents, concepts, methods, goals and context. To extract the relevant data for each dimension and standardise the focus among the research team, questions to facilitate the process were developed (see Table 3).

**Table 3:** Cultural adaptation

The ecological validity framework	
1. Language: Does the study report the use of a culturally appropriate language, idioms, regionalism words, and slang in both written and verbal forms while adopting/screening for autism?	
2. Persons: Does the study highlight ethnic and interactional match considerations between the clients and assessors in the screening process?	
3. Metaphors: Does the study employ any verbal (e.g., folk sayings) and/or visual forms (e.g., image, figure) of symbols that are shared with the population, while adopting instruments/screening for autism?	
4. Contents: Does the study consider adapting the instruments’ content to match the uniqueness culture of the study group?	
1. Concepts: Does the study present any efforts to adapt clear and consistent constructs to the targeted culture?	
2. Goals: Are the screening goals constructed within the context of cultural values, customs, and traditions?	
3. Methods: Do the study methods facilitate smooth implementation for screening within the client’s cultural context?	
4. Context: Does the study consider the social, economic, historical, and political contexts of clients while screening?	

Author	Language	Persons	Metaphors	Content	Concepts	Goals	Methods	Context
Albores-Gallo et al. [50]	+	–	–	–	+	–	–	–
Ben-Sasson and Carter [13]	+	+	–	–	+	–	–	–
Beuker et al. [51]	+	–	–	–	–	–	–	–
Canal-Bedia et al. [53]	+	–	+		+	+	–	–
Carakovac et al. [56]	+	–	–	–	+	–	–	–
Fombonne et al. [49]	+	–	–	–	–	–	–	–
Kamio et al. [46]	+	–	–	–		–	–	
Kamio et al. [47]	+	–	–	–	+	–	–	–
Kara et al. [57]	+	–	–	–	–		+	+
Kondolot et al. [55]	+		–		+		+	+
Mohamed et al. [43]	+	–	–	–	–	–	–	–
Mohammadian et al. [45]	+	–	–	–	–	–	–	–
Nygren et al. [54]	+	–	–	–	+	–	+	–
Perera et al. [53]	+	–	–	–	–	–	+	–
Perera et al. [19]	+	+	+	–	+	–	–	–
Samadi and McConkey [7]	+	–	–	–	+	–	–	–
Seif Eldin et al. [42]	+	–	–	–	–	–	–	+
Seung et al. [48]	+	–	–	–	+	–	–	–
Wong et al. [44]	+	–	–	+	–	–	+	–

The third form highlighted the aspects of feasibility that included studies may have reported and or considered while screening for autism. For that, the researchers adapted recommendations to facilitate investigation and provide a comprehensive understanding of screening feasibility [39]. These recommendations also included eight dimensions that focused on accessibility, demands, implementation, practicality, adaptation, integration, expansion, and limited efficacy. Similar to the cultural adaptation form, a questionnaire was developed to assess each dimension individually (see Table 4). Relevant information concerning cultural adaptation and feasibility was extracted by TAM and DW independently. Disagreements were encountered between the two reviewers on a few occasions but were resolved through discussion.

**Table 4:** Feasibility

The feasibility of screening	
1. Acceptability: Do study’s participants perceive an appropriateness or suitability for screening for ASD within the intended culture and context?	
2. Demand: Do study’s participants express a need and/or intention to use the screening instrument within current practice?	
3. Implementation: Was the screening process implemented as proposed?	
4. Practicality: Does the study report the cost, time and other resources required to screen for ASD?	
5. Adaptation: Does the study adapt the screening instrument for the intended population culture?	
6. Integration: Does the study highlight the possibility of integrating the screening instrument within the existing system?	
7. Expansion: Does the study perceive any opportunity to expand the use of screening within a different population in a different setting?	
8. Limited efficacy: Does the study report limited efficacy of the screening and/or its instruments?	

Author	Acceptability	Demand	Implementation	Practicality	Adaptation	Integration	Expansion	Limited efficacy
Albores-Gallo et al. [50]	–	–	+	+	+	+	+	+
Ben-Sasson and Carter [13]	–	–	+	–	+	+	+	+
Beuker et al. [51]	–	–	+	+	+	–	+	+
Canal-Bedia et al. [53]	+	–	+	+	+	+	–	+
Carakovac et al. [56]	–	–	+	+	+	+	–	–
Fombonne et al. [49]	–	–	+	+	+	–	+	+
Kamio et al. [46]	–	–	+	+	+	+	+	+
Kamio et al. [47]	–	–	+	+	+	+	–	–
Kara et al. [57]	–	–	+	+	+	–	–	+
Kondolot et al. [55]	–	–	+	+	+	+	–	–
Mohamed et al. [43]	–	–	+	+	+	–	–	–
Mohammadian et al. [45]	–	–	+	–	+	–	–	–
Nygren et al. [54]	–	+	+	+	+	+	–	+
Perera et al. [53]	–	–	+	–	+	+	–	–
Perera et al. [19]	–	–	+	+	+	+	–	–
Samadi and McConkey [7]	–	–	+	+	+	+	–	+
Seif Eldin et al. [42]	–	–	+	+	+	+	+	–
Seung et al. [48]	–	–	+	+	+	–	+	+
Wong et al. [44]	–	–	+	+	+	+	+	+

+ Data reported, −  data not reported

### Quality assessments

The selected studies were critically appraised through the use of a straightforward validated assessment tool, known as ‘QUALSYST’ [40]. This tool comprised 14 items and each study was scored, in terms of the degree to which it met the criteria of the item. The results were reported as “yes” = 2, “partial” = 1, “no” = 0. It was also possible to score a particular study design as ‘not applicable’ (“n/a”), which would exclude it from the calculation of the total score. The maximum total score is 28. The total score of rated items was then divided by the total possible score, to produce a percentage value for each paper. Using this tool the quality of studies included in was assessed by two reviewers independently (TAM and DW). Disagreements were identified between the two reviewers on scoring two papers’ methods and outcomes. Both reviewers examined studies critically and discussed quality scores until agreement was reached. No study were excluded due to study quality as all studies were scored above 0.6 which seen as an acceptable level for inclusion in a systematic review [40] (see Table 5).

**Table 5:** Quality assessments of the included studies

Authors	Albores-Gallo et al. [50]	Ben-Sasson and Carter [13]	Beuker et al. [51]	Canal-Bedia et al. [53]	Carakovac et al. [56]	Fombonne et al. [49]	Kamio et al. [47]	Kamio et al. [46]	Kara et al. [57]	Kondolot et al. [55]
Quality assessment criteria										
1. Question/objective sufficiently described	2	2	2	2	2	2	2	2	2	2
2. Study design evident and appropriate	2	2	2	2	2	2	2	2	2	2
3. Method of subject/comparison group selection or source of information/input variables described and appropriate	2	2	2	2	1	2	2	2	2	2
4. Subject (and comparison group, if applicable) characteristics sufficiently described	2	2	2	1	1	1	1	1	1	2
5. If interventional and random allocation was possible, was it described?	N/A	N/A	N/A	N/A	N/A	N/A	N/A	N/A	N/A	N/A
6. If interventional and blinding of investigators was possible, was it reported?	N/A	N/A	N/A	N/A	N/A	N/A	N/A	N/A	N/A	N/A
7. If interventional and blinding of subjects was possible, was it reported?	N/A	N/A	N/A	N/A	N/A	N/A	N/A	N/A	N/A	N/A
8. Outcome and (if applicable) exposure measure(s) well defined and robust to any measurement/misclassification bias. Means of assessment reported	2	2	2	2	2	1	2	2	2	2
9. Sample size appropriate	1	1	1	2	1	1	1	1	2	2
10. Analytical methods described/justified and appropriate	2	2	2	2	2	2	2	2	1	1
11. Some estimate of variance is reported for the main results	1	1	1	1	1	1	1	1	1	1
12. Controlled for confounding	2	N/A	N/A	N/A	1	2	N/A	N/A	N/A	N/A
13. Results reported in sufficient detail	2	2	2	2	1	1	2	2	2	2
Total sum = (number of “yes” *2) + (number of “partials” * 1)	18	16	16	16	14	15	15	15	15	16
Total possible sum = 28 − (number of “N/A” * 2)	22	20	20	20	22	22	20	20	20	20
Summary score: total sum/total possible sum	0.82	0.80	0.80	0.80	0.64	0.68	0.75	0.75	0.75	0.80

Authors	Mohammed (2012)	Mohammadian et al. [45]	Nygren et al. [54]	Perera et al. [53]	Perera et al. [19]	Samadi and McConkey [7]	Seif Eldin et al. [42]	Seung et al. [48]	Wong et al. [44]
Quality assessment criteria									
1. Question/objective sufficiently described	2	2	2	1	2	2	2	2	2
2. Study design evident and appropriate	2	2	2	2	2	1	2	2	2
3. Method of subject/comparison group selection or source of information/input variables described and appropriate	2	1	2	1	2	1	1	2	2
4. Subject (and comparison group, if applicable) characteristics sufficiently described	2	2	2	1	2	2	1	2	2
5. If interventional and random allocation was possible, was it described?	N/A	N/A	N/A	N/A	N/A	N/A	N/A	N/A	N/A
6. If interventional and blinding of investigators was possible, was it reported?	N/A	N/A	N/A	N/A	N/A	N/A	N/A	N/A	N/A
7. If interventional and blinding of subjects was possible, was it reported?	N/A	N/A	N/A	N/A	N/A	N/A	N/A	N/A	N/A
8. Outcome and (if applicable) exposure measure(s) well defined and robust to any measurement/misclassification bias. Means of assessment reported	2	2	2	1	2	2	0	2	2
9. Sample size appropriate	2	1	2	1	1	1	1	1	2
10. Analytical methods described/justified and appropriate	1	2	2	1	1	2	2	1	2
11. Some estimate of variance is reported for the main results	2	2	2	1	2	2	1	2	1
12. Controlled for confounding	N/A	N/A	N/A	N/A	1	N/A	2	N/A	2
13. Results reported in sufficient detail	2	2	2	1	2	2	2	2	2
Total sum = (number of “yes” *2) + (number of “partials” * 1)	17	16	18	12	17	15	14	16	19
Total possible sum = 28 − (number of “N/A” * 2)	22	20	20	20	22	20	22	20	22
Summary score: total sum/total possible sum	0.77	0.80	0.90	0.60	0.77	0.75	0.64	0.80	0.86

* Yes (2), Partial (1), No (0), NA = Not applicable for this study design

## Results

### Search outcomes

**Fig. 1:**
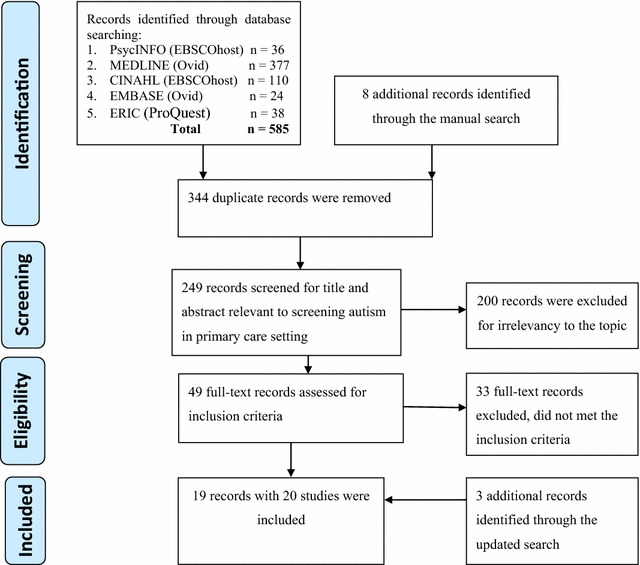
Selection process using PRISMA 2009 flow diagram

The database search yielded 585 papers and an additional eight papers were retrieved from the reference list search. Removed were 344 papers, which were duplicates, leaving 249 papers. Paper titles and abstracts were scrutinised for relevance by two reviewers (TAM, CM) and 49 papers were retained. The full text of these 49 papers was examined and checked against the inclusion criteria by the same two reviewers. Thirty three papers were excluded for illegibility. Three more papers that met the inclusion criteria for the second search were included, whereupon a total of 19 papers were selected. The updated search revealed three more studies, culminating in the inclusion of 19 papers as part of this review (see Fig. 1).

### Study characteristics

This review presented 19 papers, which included 20 studies, as one paper reported on two different studies [41]. These studies, from 13 nations (Arab [42, 43], Hong Kong [44], Iran [7, 45], Israel [13], Japan [46, 47], Korea [48], Mexico [49, 50], Norway [51], Sir Lanka [19, 52], Spain [53], Sweden [54], Turkey [41, 55] and Serbia [56]), met the inclusion criteria for this review.

All studies used observational design: cross sectional (n = 5), case control (n = 9) and cohorts (n = 6). The last 6 years (2012–2017), have seen an increased interest in autism screening, as 16 of the studies included in this review were from that period, compared with four studies from between 2004 and 2011. The study sample size varied from 100 [45] to 12,984 [51], with a mean of 2207. This sample included both sexes, aged from 1 to 13 years, with a mean age of 2.6 years. The majority of studies (n = 15) were conducted in clinical settings (e.g., primary, psychiatry and hospital) [19, 43, 45–47, 51, 53–56]. Two studies were done in the community (day care, kindergarten, preschool centres and public primary schools) [7, 13], while three studies consisted of a combination of settings.

Almost 80% of the studies used the Modified Checklist for Autism in Toddlers (M-CHAT n = 15), as well as its revised version, with the follow-up interview (M-CHAT R/F; n = 1) as a screening instrument. However, other screening instruments, such as the First Year Inventory (FYI) in Israel [13], Social Responsiveness Scale-Preschool (SRS) in Mexico [49], Quantitative Checklist for Autism in Toddlers (Q-CHAT) in Iran [45], and Pictorial Autism Assessment Schedule (PAAS) were also recognised in this review.

Parents were the main informants in all studies, especially mothers, although in some cases (n = 6) a trained assessor, such as a medical/health science student, nurse, family physician or psychologist was also involved. The trained assessor involvement was for validation purposes, or to meet cultural preferences [44, 47, 48, 53–55, 57, 58]. Training (seminars, workshops and a special study module) or aids (pamphlet slides, oral presentations, instruction booklets) were used to promote awareness of autism among both professionals and parents. However, the nature of implementation, training programmes, assessors’ roles, and detailing such awareness varied from author to author and was not fully documented.

### Cultural adaptation

The EVF model [33] was used to investigate the extent of cultural adaptation within the study. This model suggested addressing eight dimensions when culturally adapting an intervention. They are explained in following sections.

#### Language

The first dimension was that of language; placing particular attention on presenting clear and understandable language, idioms, regional words and slang, in both written and verbal forms. In this review, all studies undertook language adaptation. Each study attempted to present culturally appropriate language (verbal and written) as part of their adopted instrument, as well as in the follow-up interview. Despite similarities in linguistic adaptation procedures, studies varied in the way findings were reported. Only two studies detailed, in full, the steps involved in linguistic adaptation, such as translation, back translation, number of translators, piloting and committee review. Discussions were supported with examples [48, 53]. Seventeen studies reported some of the previous steps, most commonly, the back and forth translation [7, 13, 19, 41–47, 49–52, 54–56]. However, the translation procedure and cultural adaptation guidelines, if indeed any such guidelines were adopted, were not discernible. The exception being Nygren et al. [54] who highlighted information regarding the use of recommended guidelines for translation. In addition to translation, Seif Eldin et al. [42] incorporated different dialects from nine Arabic countries. This information was integrated into the adapted version of the Modified Checklist for Autism in Toddlers (M-CHAT), in order to promote parental understanding of autism in those countries. Perera et al. [19] attempted to conceptualise screening items in their original language, then combined each item with a photograph to facilitate parental comprehension. This step was followed by a clarity check from a random sample of professionals and members of the public.

#### Metaphors

This dimension addressed incorporating verbal (e.g., folk saying) and visual symbols such as images, pictures, or figures in the screening process to convey a meaning tailored to the cultural values. In this review, two studies used culturally relevant metaphors within the screening process. For example, Canal-Bedia et al. [53] developed a Spanish version of the M-CHAT and, after piloting, included an adaptation, using Spanish cultural idiosyncrasies. Items 3, 5 and 23 were modified to include examples of Spanish toys. Perera et al. [19] introduced photographs within their screening instruments, to illustrate the text of the screening items and to promote understanding.

#### Person

This dimension addresses the ethnic or interactional match between study participants and the investigator. “Person” was only considered on two occasions. The first was in Ben-Sasson and Carter [13], where only parents who were proficient in Hebrew were involved, which enabled them to complete the adapted version of FYI in the Hebrew language and culture with more ease. Perera et al. [19] used local children’s photographs to promote parental understanding of the screened items.

#### Contents

In some studies, the culture, values, customs and traditions of participants were integrated into the content of the adapted instruments and/or screening process. Only one study investigated the value of cultural information from the study groups and incorporated that into the screening instrument. Specifically, Wong et al. [44] modified the original instrument, from a checklist format to a graded score system, as a result of a pilot study which found many Chinese parents struggled to answer the original yes/no questions. The modified CHAT-23 involved selecting answers, such as “never”, “seldom”, “usually,” and “often”.

#### Concepts

Ten studies described how the authors’ framed the adapted instruments into formats that were more understandable and consistent with the specific culture and context. This involved re-wording, describing and generalising/specifying difficult concepts or supporting them with clarifying examples, in a written or demonstrable format, or by deleting confusing, less well understood items. For example, in Canal-Bedia et al. [53] three items from the screening instrument were re-worded after piloting to promote parental understanding (items 5, 8, and 17). Albores-Gallo et al. [50] described the meaning of the “peek-a-boo” game because some parents, such as Mexican parents, did not have a name for it. In Kamio et al. [47] and Kondolot et al. [55], trained interviewers provided parents with specific examples for each failed item, to facilitate a better understanding and enable them to judge their responses. Samadi and McConkey [7] provided a general definition for some items, when translated to the Kurdish and Persian languages, to promote parental understanding. For example, item 9, “finger flicking,” was presented in the Kurdish instrument as “any unusual finger and hand movements.” Item 10, ‘fearful behaviours’, was explained during the follow-up interview, as reactions to social situations and new experiences. Seung et al. [48] also re-worded three items (3, 5 and 11), and included examples for each and deleted the three most confusing and misunderstood items (4, 8 and 22). More explicit words for a number of unspecified items were also included, to promote instrument adequacy and an understanding for Korean parents, consistent with Nygren et al. [39], who used interpreters to describe items 11, 22, and 23. Perera et al. [19] incorporated relevant photographs within their study, to clarify item concepts and improve recognition.

#### Goals

From the studies reported here, it was not possible to identify whether the screening goals were constructed within the context and knowledge of values, customs and traditions, or if there was any similarity among the assessors and participants in terms of screening being desirable within the study context. This is with the exception of one study in Spain, in which the authors reported that both professionals and parents expressed an interest in routine autism screening [53].

#### Methods

Methods takes into consideration the incorporation of cultural knowledge into screening methodology. Five studies incorporated cultural knowledge and modified screening methods to ensure screening falls within that country’s cultural context. For example, a study by Kara et al. [41], found that when Turkish parents filled in the M-CHAT [59], 49% of participants’ screened were positive for autism. As a result, in the second study trained nurses and psychologists interviewed parents to completed the Turkish version of the M-CHAT questionnaire, where they were able to probe and clarify issues. This method proved more effective and followed a recent study [55] where the M-CHAT [59] was completed using information gathered in face-to-face interviews. This, again, was found to be useful in the Turkish culture and resulted in fewer false-positive screening results. Another example of methodological modification to meet cultural preferences and improve instrument reliability, was in a study by Wong et al. [44], where Chinese parents did not complete the entire questionnaire checklist. An observational section, completed by a trained assessor, was found to reduce false-positive results. For the same purpose, other studies incorporated the screening instrument, M-CHAT, with different instruments [52, 54], or with a follow-up interview, to enhance reliability and meet cultural needs.

#### Context

Context is the last dimension of the framework and takes into account the contextual issues that may affect the screening process within each culture. This review found authors of the described studies attempted to address issues which might have challenged autism screening and they suggest potential efforts to overcome these challenges. For example Kara et al. [41], Kondolot et al. [55], identified a context issue among the Turkish population: the general population was not used to completing checklists and, hence, preferred verbal interview formats. Low and middle-income families in Turkey may also have difficulty in understanding the written questionnaire. The number of years spent in education is lower (not specified) in Turkey than in Western nations. Seif Eldin et al. [42] produced an Arabic version of the M-CHAT to screen children for autism in nine Arabic countries. Participating countries were classified into four sub-groups (the Gulf area, East Mediterranean, Egypt and Tunisia) based on cultural, ethnic, political and social structure similarities, in order to reduce the impact of cultural diversity and help generate concrete conclusions. However, the authors did not report how they accounted for other cultural influences.

### Feasibility

In Bowen et al. [39] taxonomy of feasibility constructs were used to evaluate feasibility aspects for each study. Some information relevant to these aspects was identified and detailed in the following sections for each dimension.

#### Acceptability

With the exception of one study, the perception of suitability or satisfaction towards autism screening was not documented. In their two-phase study, Canal-Bedia et al. [53] adapted and validated the M-CHAT for the Spanish population, highlighted the “great interest” that both parents and professionals showed in routine screening for communicative and social development in Spain.

#### Demand

Only one study documented interest in using autism screening within their current practice. Nygren et al. [54] trained doctors and nurses in child health care settings to screen children for autism, within the two and a half years of age check-up window. The study highlighted that the trained assessors continued to use their newly acquired skills to refer suspected cases of autism (in children both younger and older than two and a half years) for evaluation, even after completion of the study.

#### Implementation

Although the studies varied in design, purpose and results, screening for autism seemed to be successfully implemented, as planned, for the intended participants. However, the studies investigated here varied in the detail of the implementation process. Five studies provided full detail of the planning and implementation process associated with screening [13, 44, 48, 51, 53]. The remaining studies briefly explained what they had undertaken [7, 19, 42, 43, 45, 46, 49, 50, 52, 55, 57]. The shortened explanations might be the result of journal word limits.

#### Practicality

Most studies reported that screening instruments identified autism, but expressed concern over their adequacy in population-based settings. Studies also highlighted the cost burden of vetting instruments [13, 43, 46, 49–51, 53] and the interventions required to redress limitations, like training assessors and employing follow-up interviews [50, 55]. Among all screening instruments, the M-CHAT and revised versions, including follow-up interviews, were adopted by almost 80% (n = 16) in the studies reviewed. M-CHAT was implemented either separately or with another instrument (Checklist for Autism in Toddlers (CHAT), Early Screening of Autistic Traits Questionnaire (ESAT), Joint Attention Observation (JA-OBS), CBCL/15.5-5 Hiva and/or a follow-up interviews) for cultural preferences or validation purposes. Despite the disparity in implementation, analysis and adaptation methods, similarities were noted in the practical features of M-CHAT across numerous studies (i.e., time and key identifers). For example, studies reported that the M-CHAT can be completed either by a parent or by an assessor within 5–10 min and the follow-up interview would need a further 10 min. Interestingly item 13 “imitate you” was found to be the only key identifier item from the original M-CHAT (i.e., can discriminate between children with or without ASD) across nations, with some variation in strength for the identification of autism. The reviewed studies also presented the differences in other key identifer items from the original M-CHAT, such as item 21 “understanding” [41, 46, 47, 50, 53, 54], and item 23, “checking reaction” [44, 46, 47, 53, 54]; while item 11, “over-responsiveness to noise” presented some concerns in five studies [41, 43, 48, 53, 54].

Besides M-CHAT, this review identified other instruments that lent themselves to being completed by parents in a short time frame. For example, the First-Year Inventory (FYI) includes 60 items, takes about 20 min to rate the 60 items as: never, seldom, sometimes and often, or includes multiple choice questions to identify children at risk of autism or a related developmental disability. Similarly, SRS, a 65-item rating scale, ranging from 1 (not true) to 4 (almost always true), requires 15–20 min to complete. In contrast, Quantitative Checklist for Autism in Toddlers (Q-CHAT), scored on a 5-point scale (0—never to 4—always) contains 25 items, and takes 5–10 min to complete. Finally, the 21 PAAS items with “yes” or “no” choices, can be completed in 15–20 min.

#### Adaptation

Adaptations were made in all studies, with variations to accommodate cultural values and traditions, depending on the study aims and perspective.

#### Integration

Integrating the screening process into an existing system was common among studies but is not encouraged. The studies suggest the possibility of introducing autism screening at the primary level (paediatric, surveillance programme and routine practice) [13, 42, 44, 46, 47, 50, 52–54, 56], psychiatric level [50] or within a school setting [45]. However, they also warned of potential instrument inadequacy, as well as any cultural or demographic influences on the screening context. Some studies also noted the importance of recognising individual health system needs and capacities, prior to introducing mandatory screening programmes [44, 52, 53, 55, 56].

#### Expansion and limited efficacy

Most studies did not encourage autism screening beyond the study context and indicated limited efficacy in adapting the instruments for different populations. Results of studies varied, making it very difficult to compare them internationally and formulate conclusions. For example, studies adapted various screening instruments (M-CHAT, M-CHAT R/F, Q-CHAT, CHAT-23, SRS, FYI and PAAS) that represented diverse levels of psychometric properties (i.e., reliability and validity of the instrument) [13, 19, 44, 49, 51, 54]. Even within studies that used the same instrument (M-CHAT), variations of responses to the instrument items, key identifiers [42, 44, 48, 50, 54], and instrument adequacy were reported [13, 46, 50, 51].

## Discussion

Nineteen papers (incorporating 20 studies), from different geographical regions were included in this review, aiming to determine the level of content adaptation that was considered when adapting instruments to screen for autism in non-English speaking countries. The review also highlighted feasibility aspects of screening for autism in the countries included, if any were reported. Despite variation in description and documentation of the investigated points, there were some commonalities across findings which helped the reviewers to draw relevant conclusions. They are explained in following sections.

### Surface versus deeper level of adaptation

In this review, it was clear that most of the studies used surface modifications, the main focus being translation, with only a few studies also implementing deeper level adaptations. Various steps and measures were undertaken to ensure verbal and written language used in the screening process was clear, understandable, culturally appropriate and syntonic to the individual culture. However, the authors concurred with Soto et al. [29], in that little information was offered to enable conclusions to be drawn on how such adaptations were maintained or guided. For example, the majority of studies mainly reported back and forth translations. Other aspects of surface modifications (e.g., metaphors) were less recognisable, and/or reported in the reviewed studies.

Translation is the first step involved in the adaptation of an instrument. It requires careful planning and equal treatment of linguistic, cultural, contextual and scientific information [60, 61]. Yet, despite the significance of this step, some authors failed to report basic details, such as how many translators were involved and what their qualifications were. Recent evidence indicates the need for a minimum of two bilingual translators, with a cultural background and proficiency in both languages, to minimise the risk of linguistic, psychological, cultural and understanding (i.e., theoretical and practical) biases [60, 62]. Some studies failed to include an expert review or a pilot study. Both steps are essential in synthesising the suitability of an instrument for the targeted cultural context or in approving its readiness for use.

On the other hand, deep levels of adaptation were noted in a few studies throughout the following EVF domains: concepts, contents, methods and context, to redress some cultural and comprehension issues. Among them, the most commonly used domain was adaptation of concepts. In this domain, authors reported efforts to re-word some items in their instruments, using more culturally sensitive concepts to screenings, excluding confusing or difficult items, or presenting participants with clarifying examples framed within the investigated cultural values and traditions [13, 48, 50, 51, 53, 54]. This was followed by an adaptation in methodology that required the researcher to change the screening instrument methodology from a parental report checklist to a trained assessor or interview format. This type of adaptation was undertaken to improve the rigorous nature of the instrument [7, 44, 47, 52, 54]. The least considered domains of deeper adaptation were context and contents. Very few studies considered incorporating information on cultural value, such as the level of education, socio-economic status or the geographic and demographic characteristics of the population [41, 42, 44, 48].

Reviewed studies lacked the justification for favouring a surface type of cultural adaptation. This might be due to the absence of available information on autism screening in each contexts. However, the body of research on autism screening is growing, rapidly and globally. Future studies might be able to identify the level of cultural adaptation and the resources required beforehand. The lack of feasibility studies in this area might be another reason researchers were prevented from conducting deep-level cultural adaptation investigation. This may be due to an inability to estimate expected expenses and required resources for this level of adaptation. In addition to the lack of data on the practicalities of implementing autism screening and the acceptability of screening in the targeted population, cost effectiveness analysis and random controlled trial studies, comparing the satisfaction levels of autism screening groups with that of control groups, might be valuable in advancing this area. Lack of investigator knowledge, interest and expertise on cultural adaptations might be another reason for inadequate documentation and justification for adapting a screening instrument.

Additionally, studies in this review lacked details on the particular cultural adaptation framework that was followed, as well as the efforts taken to avoid bias. This issue was also revealed by Baumann et al. [34], who advocated for an unambiguous description of what had been adapted, why it had been adapted, and how it was adapted. Adequate reporting is necessary for future studies, to promote an effective outcome, maintain high fidelity and avoid decrements in screening impact [34]. The literature also provides a number of guidelines to ensure adequate adaptation is achieved at the process [24, 60, 62, 63] and/or content levels [33, 64]. Noting such guidelines and integrating them within the screening process, may reduce discrepancy among results and enable researchers to replicate studies, and investigate differences between instruments within an increasingly diverse population [62].

### Aspects of feasibility on screening for autism

With advances in knowledge of autism screening, identifying the feasibility of this programme has become essential in minimising resource waste, in prioritising decisions and in improving the strength of health care organisations [31]. The studies investigated as part of this review, are generally concerned with the practicalities of screening instruments, in terms of their adequacy, time, cost, and training required to deliver effective screening.

In non-English speaking countries, M-CHAT was a popular screening instrument [7, 41–44, 48, 51, 53, 55, 56], albeit with a number of language and cultural adaptations, as discussed earlier. Adaptation suggests an effectiveness in improving instrument properties (sensitivity, specificity and PPV values), reducing false identification and unnecessary burden. However, this raises concerns regarding costs of training staff and allocating follow-up interviews for parents, especially in those countries with limited staff and resources.

Responding to global use of M-CHAT, a new version of this instrument with 20 items, referred to as a Modified Checklist for Autism in Toddlers, Revised with Follow-Up (M-CHAT-R/F) [65], was released. Despite the existence of this version, all studies in this review, with the exception of a recent one [56] adopted the original version of the M-CHAT, with 23 items. Adopting the new version might reduce the challenges of dealing with some difficult items (i.e., 1 and 4) [43, 50, 53] and providing supporting examples to reduce any future misunderstanding and to improve instrument properties. Carakovac et al. [56] reported less positivity and improved results, when compared with previous M-CHAT studies. Using the revised version of the M-CHAT in future studies might improve its practicality.

Previous M-CHAT studies attributed cultural impacts for any inconsistencies between item responses. However, recent evidence revealed additional reasons, such as demographic characteristics. For example, the level of education might reduce a parents’ ability to understand items in the questionnaire [41, 50, 52]. A lack of parental comprehension might also be the result of parents sharing some autistic characteristics with the child, making it difficult to recognise the abnormal signs of autism [50]. This, therefore, could reduce their credibility, as the sole informants for autism screening and might explain the improved result achieved when a trained assessor or follow-up interview was incorporated into the parental self-report in M-CHAT studies. Another potential reason for discrepancy was the problem of reversed coding for certain items in the M-CHAT, such as items 11, 18 and 22 [48]. Seung et al. [48] recommended adapting and using these items with caution. To improve the practicality of adapting the M-CHAT, issues like these should be considered and investigated, to avoid wasting time, effort, and resources.

Reducing the number of items in other screening instruments found in this review (SRS and FYI) might help speed up the screening process, facilitate its integration into a busy clinical setting, promote the cooperation of parents and make it easier for both parents and professionals, with limited experience, to comprehend the questions and complete them with ease. There appears to be a movement towards the development of screening instruments with less items (e.g., 10 items). The studies have identified the most definitive items that would accurately pinpoint a symptom of autism [7, 11, 46] and proposals to increase their use in screening instruments in the future. Incorporating visual aids (i.e., photographs or pictures) and conceptualising the instrument items using the original language, as was the case in Perera et al. [19], may potentially facilitate a parent’s comprehension and reduce cultural as well as adaptation barriers.

It was evident recent screening scales are moving towards quantitative measurements, with items reorganised as Likert scale types. This was established on the assumption autistic traits normally are distributed in the general population, not only in parents, but also in individuals with no previous diagnosis of autism in their families [50]. Despite advancing knowledge in this area and promising results, these abbreviated quantified instruments warrant further validation globally, considering a participant’s characteristics, such as social factors, cognitive level, and medical history [66]. This will enrich our understanding of the factors that might influence the accuracy of the instruments from a global perspective.

Due to limitations in the screening instruments, scholars have not encouraged integrating autism screening within routine practice and warranted further investigation for individual cultures. For the same reasons, expanding the screening programme beyond the study context and for different populations was not favoured by most researchers, as it indicated limited efficacy.

Cost effectiveness on autism screening is an important practicality to consider when introducing a new programme [39]. However, research in the field of autism screening cost is limited. Assessing the costs of screening might provide a comprehensive insight into the eventual financial burden of both direct (e.g., medical expenditures) and indirect, (e.g., special education/training services, lost productivity by family caregivers [66], parental stress and the hassle of following positive screen participants) factors. Future research is recommended, to adequately compare various screening strategies and potentially identify, the most cost-effective methods for each individual study context. Countries vary significantly in their medical facilities and services. Regions with limited capacity for mental health assessment and services should ensure adequate resources, the sufficient coordination of services in the primary sector and early intervention prior to introducing any autism screening [41, 50, 53]. Future research should investigate the resources and cost effectiveness of introducing autism screening processes into clinical settings, as this will inform and direct future policy decisions. It is also noteworthy that coordination between healthcare and specialised services, in terms of follow-up, along with adequate preparation for early intervention, are crucial in enhancing the benefits of early identification of ASD.

Despite concerns regarding cost, increasing professional awareness and training of professionals to screen for autism were found to be useful in the studies reviewed here, in terms of facilitating the screening process and improving the rigorous nature of the instruments. Kondolot et al. [55] also highlighted the benefits of training staff to screen for autism and the fact it might reduce the high positivity that results from screening instruments used by inexperienced staff or parents. However, studies varied in their documentation of the training received and therefore comparisons could not be made regarding the level of training required or the expenses incurred to facilitate effective screening. Training professionals to recognise early signs of autism is recommended in the clinical guidelines [67], as without standardised training, vital signs and differences in screening results may occur.

### Ignored areas in cultural adaptation and feasibilities model

Ultimately, investigators failed to capture some common areas in both models: cultural adaptation (i.e., ‘person and goals’) and feasibility (i.e., ‘acceptability, and demands’). In most studies, participants’ interests, views, perceptions, understanding and agreement, in relation to autism screening goals and implementations, were not discussed or documented much. These areas are essential to bring acceptance to the adapted programme, reducing ethnic and racial discrepancies between investigators and participants, promoting cooperation, increasing demands for autism screening, as well as producing a flexible screening programme, framed within the values, customs and traditions of the targeted population [33]. Future studies should consider both domains when adapting screening programmes to accommodate cultural discrepancies, raising investigator credibility and improving participant and investigator relationships towards an effective outcome.

### Strengths and limitations

This review is the first to consider exploring content level of cultural adaptation and the feasibility of screening for autism in non-English-speaking countries, and therein lies its strength. It may serve as a baseline for future practitioners considering adapting an autism screening process for these populations. In terms of rigour, all stages of the process (data selection, extractions and quality validation) were cross-checked by two individuals.

Nonetheless, this review had a number of limitations. No studies written from the non-English speaking literature were included, due to limited resources for translation. The small number of identified studies represents only 13 cultures and thus has limited efficacy globally. However, important and relevant investigated aspects do emerge from the reviewed studies that may guide future work.
